# Novel Hexagonal Dual-Mode Substrate Integrated Waveguide Filter with Source-Load Coupling

**DOI:** 10.1155/2014/915740

**Published:** 2014-04-22

**Authors:** Ziqiang Xu, Gen Zhang, Hong Xia, Meijuan Xu

**Affiliations:** Research Institute of Electronic Science and Technology, University of Electronic Science and Technology of China, Chengdu 611731, China

## Abstract

Hexagonal dual-mode cavity and its application to substrate integrated waveguide (SIW) filter are presented. The hexagonal SIW resonator which can combine flexibility of rectangular cavity and performance of circular cavity is convenient for dual-mode bandpass filters design. By introducing coupling between source and load, the filter not only has good selectivity due to two controllable transmission zeros, but also has a small size by the virtue of its single-cavity structure. A demonstration filter with a center frequency of 10 GHz and a 3 dB fractional bandwidth of 4% is designed and fabricated to validate the proposed structure. Measured results are in good agreement with simulated ones.

## 1. Introduction

Dual-mode cavity bandpass filters have been widely used in the development of various wireless communication systems. The metal waveguide dual-mode filters have excellent performance owing to their high *Q* factor and power-handling capability. However, they cannot be easily integrated with microwave planar circuits [1, 2]. Recently the substrate integrated waveguide (SIW), which is synthesized in a planar substrate with arrays of metallic via, provides a low-profile, low-cost, and low-weight scheme while maintaining high performance [3–5]. Particularly, the application of SIW technology makes the implement of dual-mode cavity filters with compact size and easy integration possible [6].

On the other hand, filters with multiple transmission zeros (*TZ*s) are required to meet the increasing demands of modern communication systems in regards to compact size and high selectivity. Commonly, no more than one *TZ* can be obtained in a conventional single-cavity dual-mode filter. In order to generate more *TZ*s, many approaches have been proposed to design dual-mode SIW filters. One approach is cascading two adjacent dual-mode rectangular cavities to generate up to two *TZ*s in stop band [7]. Similarly, a dual-mode filter using two connecting circular cavities with two *TZ*s is introduced in [8]. However, their physical sizes will become larger because of cascaded structures. Another approach can be fulfilled by marshaling the effect of source-load coupling in single cavity. By adding a direct signal path between the source and the load, *N* finite transmission zeros can be generated. In [9], a dual-mode filter using a nonresonating node with indirect source-load coupling is proposed, and two *TZ*s are obtained in such a single-cavity filter.

Ordinarily, conventional dual-mode SIW filers are always built based on rectangular and circular cavities. In our previous work, a novel hexagonal resonator using SIW technology and its applications to trisection filters are proposed in [10]. The hexagonal SIW cavity can combine flexibility of rectangular cavities and performances of circular cavities. Meanwhile, as any of the six sides of a hexagonal resonator can be utilized for coupling, the filter configuration is very flexible to design. In this paper, we present a SIW filter with dual-mode hexagonal cavity. By introducing coupling between source and load, the filter not only has two *TZ*s to improve frequency selectivity, but also has a small size by profit from its single-cavity structure.

## 2. Filter Analysis and Design

**Figure 1:**
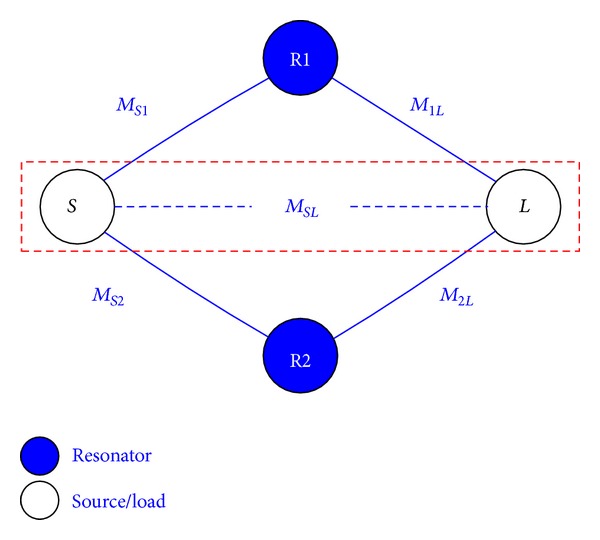
Coupling scheme of proposed dual-mode filter.

Figure 1 shows the coupling topology of the proposed dual-mode filter. By adding the coupling between the source and the load, one additional *TZ* can be obtained. In other words, the source and the load are directly coupled which can add an extra transmission path. Under this circumstance, the topology can generate up to two *TZ*s. The coupling matrix *M* of the proposed topology can be written as

(1) $$\begin{array}{c} M=\left[\begin{array}{cccc} 0 & M_{S1} & M_{S2} & M_{SL}\\ M_{S1} & M_{11} & 0 & M_{1L}\\ M_{S2} & 0 & M_{22} & M_{2L}\\ M_{SL} & M_{1L} & M_{2L} & 0 \end{array}\right] \end{array}$$

The conventional doublet without source-load coupling has a *TZ* in the stopband, and an explicit expression relating the coupling elements and the transmission zero Ω is provided in a low-pass prototype as follows [11]:

(2) $$\begin{array}{c} \Omega =\frac{M_{11}M_{S2}^{2}-M_{22}M_{S1}^{2}}{M_{S1}^{2}-M_{S2}^{2}}. \end{array}$$

Here, since the topology exhibits symmetrically, the relationships *M*_*S*1_ = −*M*_1*L*_ and *M*_*S*2_ = −*M*_2*L*_ can be hold.

When introducing the source-load coupling into this doublet, an additional *TZ* can be obtained. To get more insight of location of two *TZ*s in this topology, an explicit expression relating *M* and the *TZ*s is given by

(3a) $$\begin{array}{c} \Omega =a\pm {\left(b^{2}+c^{2}\right)}^{1/2}, \end{array}$$

where

(3b) $$\begin{array}{c} a=\frac{M_{S1}^{2}+M_{S2}^{2}}{2M_{SL}}-\frac{M_{11}+M_{22}}{2},\\ b=\frac{M_{S2}^{2}-M_{S1}^{2}}{2M_{SL}}-\frac{M_{11}-M_{22}}{2}, \end{array}$$

where *Ω* = (*ω*/*ω*_0_ − *ω*_0_/*ω*)/FBW is normalized angular frequency.

**Figure 2:**
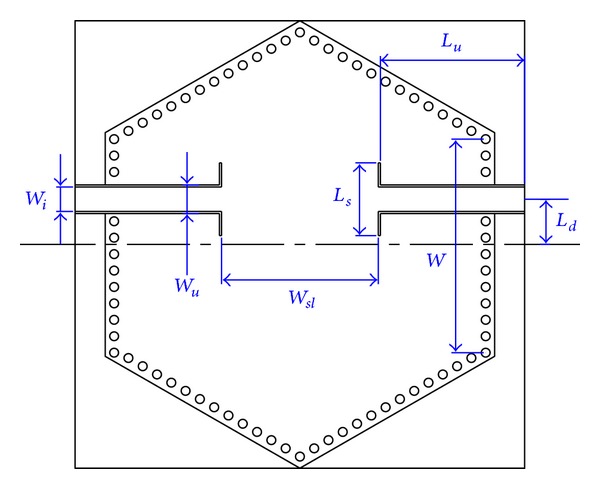
Geometric configuration of the proposed filter.

**Figure 3:**
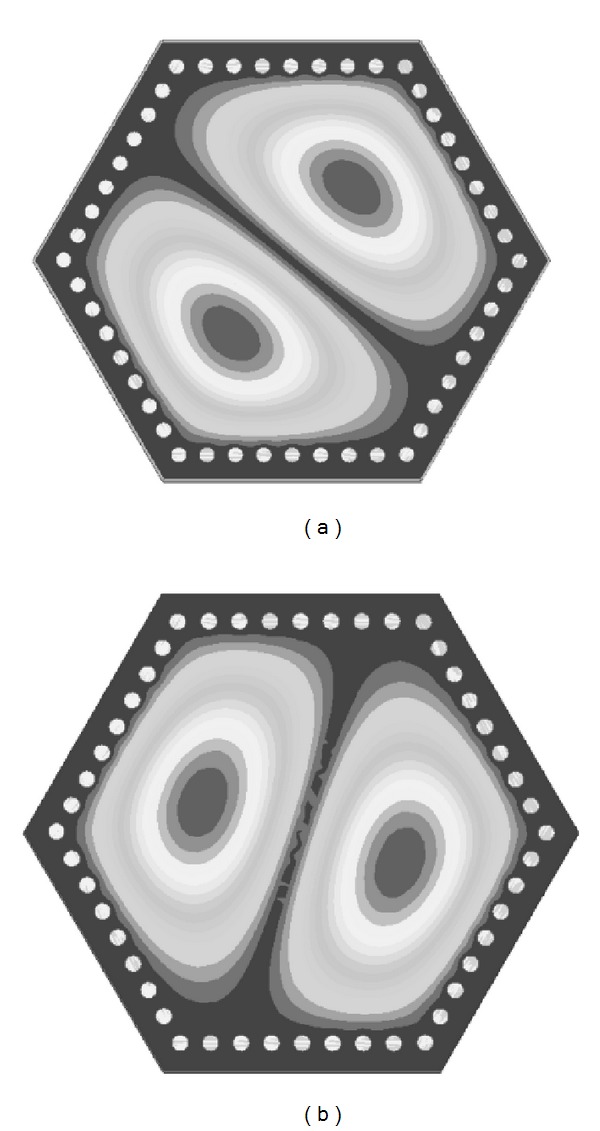
The E-field distributions of *TM*_110_ degenerated mode: (a) left inclined mode and (b) right inclined mode.

To achieve the proposed topology, a SIW filter with hexagonal dual-mode cavity is designed and embedded in a PCB substrate as shown in Figure 2. The single cavity operates with *TM*_110_ mode which consists of two intersectant modes illustrated in Figure 3. Bypass cross-couplings between the modes and source/load are introduced through symmetrical feeding structure, while source-load coupling is introduced by the up-close input and output ports.

As far as we know, there is no exacted equation for calculating the resonant frequencies through geometrical parameters in a dual-mode hexagonal cavity. According to conventional resonant frequency formulas of metallic circular waveguide resonators, the corresponding resonant frequency of *TM*_11_ in the hexagonal cavity can be determined by modified formulas as follows:

(4) $$\begin{array}{c} f_{11}=\frac{C}{\sqrt{\varepsilon_{r}}}\cdot \frac{\mu^{\prime }}{2\pi W}, \end{array}$$

where *C* is the speed of light, *ε*_*r*_ is the relative dielectric constant of dielectric substrate, *μ*′ = 4.27 is the modified root coefficient based on the Bessel function, *f*_11_ is the resonant frequencies of *TM*_11_ mode in the hexagonal SIW cavity.

**Figure 4:**
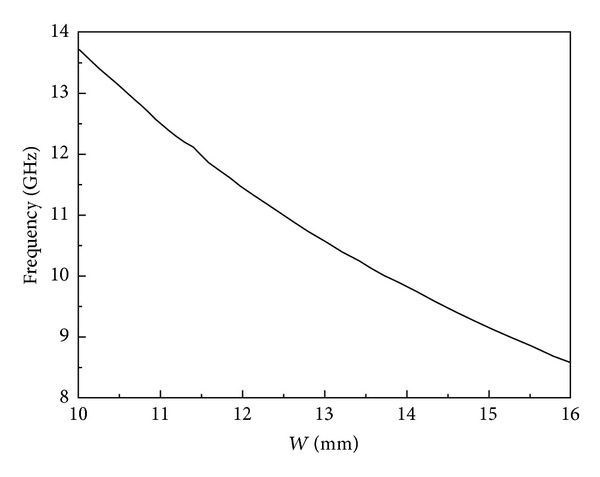
Resonant frequencies with different values of *W*.

Figure 4 shows the relationship between the fitted size and the resonant frequency of the hexagonal cavity. As can be seen, the resonant frequency of *TM*_11_ mode decreases when the geometrical parameter *W* increases.

**Figure 5:**
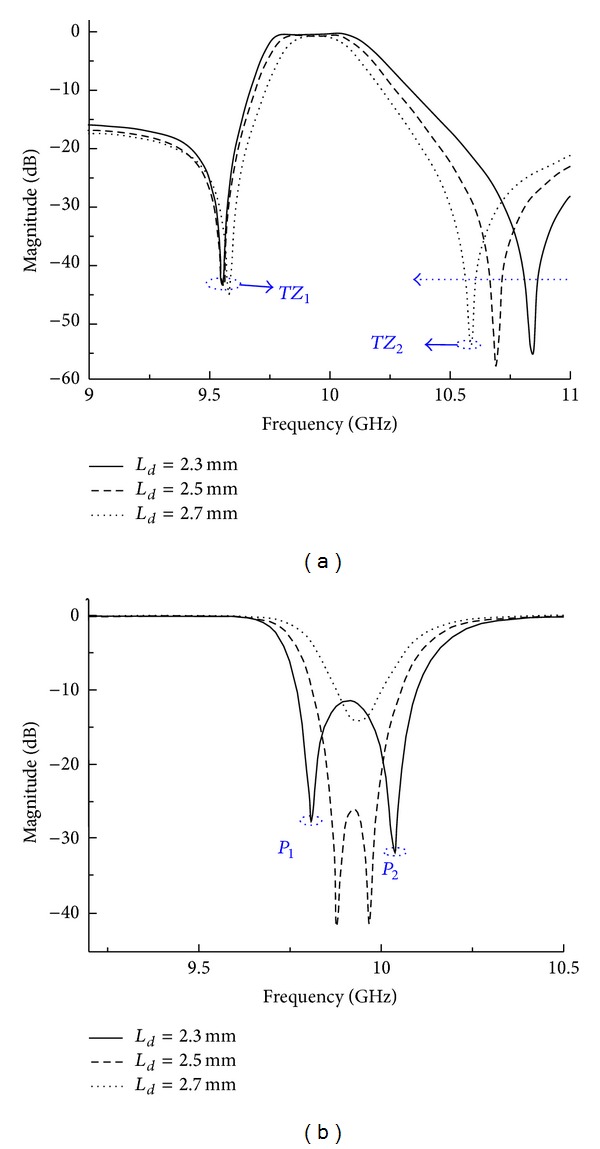
Frequency response for different values of *L*_*d*_: (a) *S*_21_ and (b) *S*_11_.

A feeding technique named current probe is adopted in the I/O SIW design to achieve the transition from SIW to microstrip. As the symmetrical input/output dominates the bypass cross-coupling, offset *L*_*d*_ between center line and feeding structure has obvious influence on the frequency response.  Figure 5 shows the frequency responses for different values of *L*_*d*_. Donate the *TZ*s at the lower and upper stopbands as *TZ*_1_ and *TZ*_2_, respectively. *TZ*_2_ is produced through bypass cross coupling; thus it move towards the lower frequencies with increasing values of *L*_*d*_. As *TZ*_1_ is dominated by source-load coupling, its location changes slightly when varying the values of *L*_*d*_. The feeding structure is right upon *TM*_110_ mode, hence positions of poles change while varying *L*_*d*_. As shown in Figure 5(b), *P*_1_ and *P*_2_ shift towards each other when the value of *L*_*d*_ increases.

**Figure 6:**
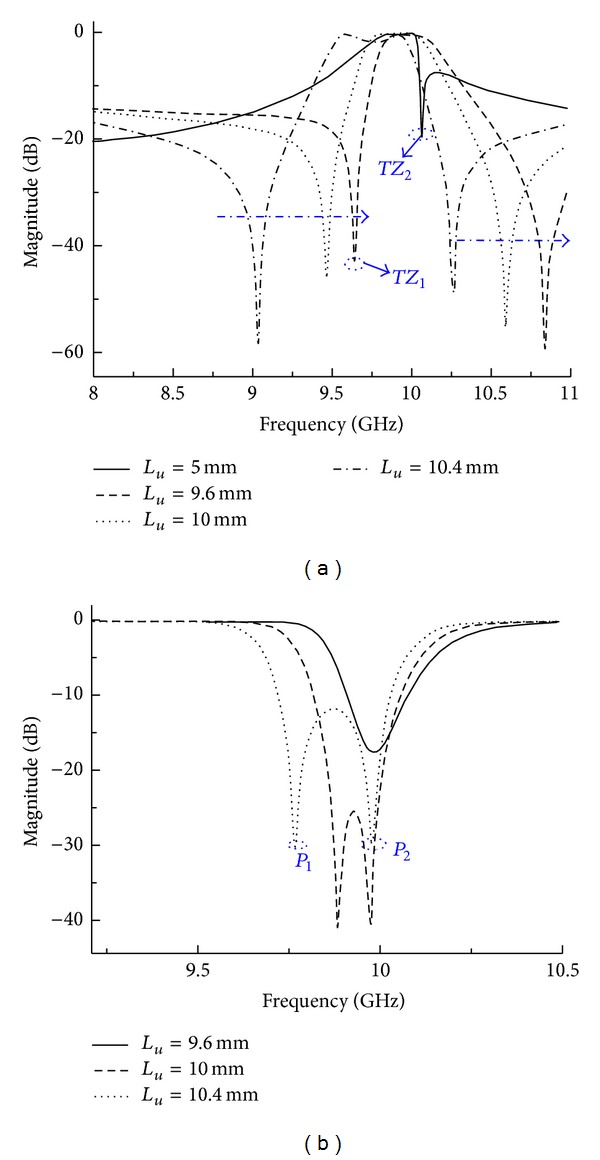
Frequency response for different values of *L*_*u*_: (a) *S*_21_ and (b) *S*_11_.

On the other hand, the length (*L*_*u*_) of input/output current probes in feeding structure determines not only the quality factor (*Q* factor) of the filter, but also the strength of source-load coupling. Frequency responses for different values of *L*_*u*_ are illustrated in Figure 6. As shown in Figure 6(a), only *TZ*_2_ at upper stopband is obtained when the value of *L*_*u*_ is too small to introduce source-load coupling (e.g., *L*_*u*_ = 5.0 mm). By increasing *L*_*u*_ to implement source-load coupling, *TZ*_1_ at the lower stopband can be realized. Then the increase of *L*_*u*_ will result in increasing of source-load coupling; hence *TZ*_1_ moves towards the passband. In fact, *L*_*u*_ is a parameter which also influences bypass cross coupling, so *TZ*_2_ shifts away from the center of the passband when the value of *L*_*u*_ increases. *L*_*u*_ has impact on *S*_11_-parameter similar to *L*_*d*_. As described in Figure 6(b), *P*_1_ shifts away from *P*_2_ and the passband broadens towards the lower frequencies when *L*_*u*_ augments. To achieve demanded frequency responses during design process of the proposed dual-mode hexagonal SIW filter, parameter of feed probes should be carefully tuned.

## 3. Experimental Results

**Figure 7:**
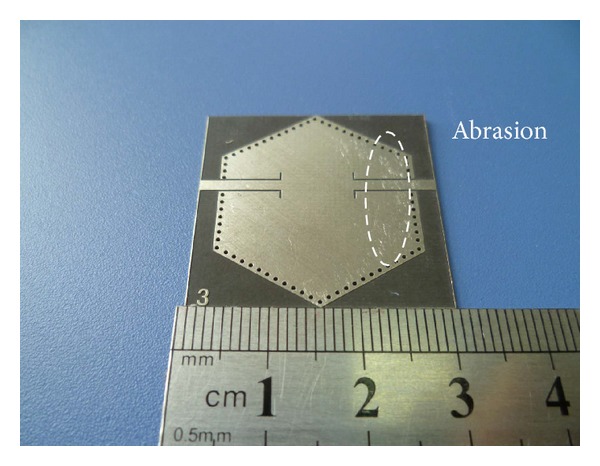
Photograph of the fabricated filter.

To validate the above-mentioned concept, a 10 GHz hexagonal SIW filter with a 3 dB fractional bandwidth of 4% is fabricated on a PCB substrate with dielectric constant of 2.2. The complete parameters are finely tuned by using commercial full wave electromagnetic (EM) simulation software HFSS. Detailed dimensions of the proposed filter are illustrated in Table 1. The photograph of the fabricated filter is shown in Figure 7. By virtue of the single hexagonal cavity and flexible source-load coupling manner, the overall size of the filter is 28.6 mm × 28.6 mm × 0.508 mm.

**Figure 8:**
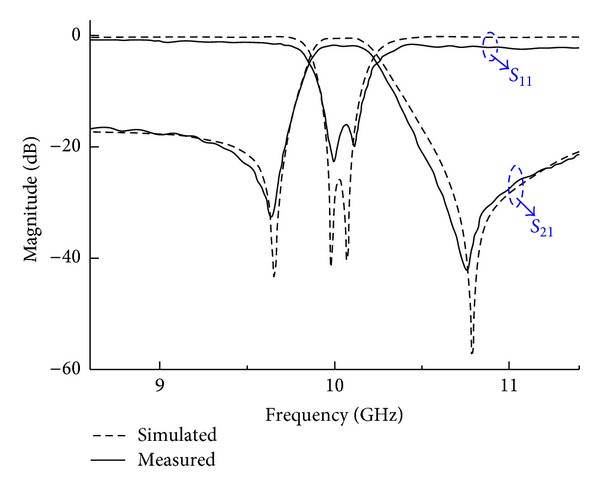
Simulated and measured results of the fabricated filter.

An Agilent E8363B vector network analyzer is used for measurement. The measured and simulated frequency responses are plotted in Figure 8. The measured result shows a central frequency of 9.99 GHz with a fractional bandwidth of 3.9%, minimum passband insertion loss of 1.66 dB, and in-band return loss greater than 17 dB. In addition, there are two transmission zeros located at 9.6 GHz with 35.6 dB rejection and 10.75 GHz with 42.5 dB rejection, respectively. The measured results are in good agreement with the simulated ones except a small frequency shift of *TZ*s and a little discrepancy in the in-band insertion loss. The degeneration of the in-band insertion loss may be caused by the test fixture as well as the abrasion on the surface. Overall, the measured results validate the feasibility of the proposed design, with its high selectivity being demonstrated.

## 4. Conclusion

A novel compact hexagonal dual-mode SIW filter with high selectivity is proposed. Two *TZ*s are produced to improve the frequency selectivity by introducing source-load coupling to the proposed single-cavity filter. A filter sample is fabricated, and the measurement results agree well with EM full wave simulation. Its compact size and high selectivity make it suitable for microwave communication application.

## Figures and Tables

**Table 1:** Parameters of the proposed filter.

Parameter	*W*	*W*_*i*_	*L*_*d*_	*L*_*s*_	*W*_*u*_	*L*_*u*_	*W*_*sl*_
Value (mm)	13.9	1.56	2.5	4.16	2.16	10.1	9.1
